# Macrophages in Glioblastoma and How Non-Coding RNAs Impact Their Differentiation

**DOI:** 10.3390/cells14191528

**Published:** 2025-09-30

**Authors:** Emily S. Westemeier-Rice, Edjah K. Nduom

**Affiliations:** Department of Neurosurgery, School of Medicine, Emory University, Atlanta, GA 30322, USA; emily.rice@emory.edu

**Keywords:** non-coding RNA, macrophage, macrophage polarization, glioblastoma multiforme

## Abstract

Within the glioblastoma (GBM) microenvironment, macrophages are widely implicated in immunosuppression. The neuro-oncology community is interested in modulating this immune suppression to improve therapies for GBM, but this approach poses significant challenges. Non-coding RNAs present an attractive target for therapeutic modulation of the immune system, and they are implicated, broadly, in macrophage polarization and the pathophysiology of GBM. Here, we discuss the extant literature on non-coding RNAs and how they impact the polarization of macrophages with particular emphasis on their impact within glioblastoma. We further discuss the clinical relevance of these non-coding RNAs and macrophage polarization to disease progression and associated therapeutic opportunities.

## 1. Introduction

Glioblastoma (GBM) is the most abundant form of brain cancer, accounting for more than half of all malignant brain tumors [1]. Affecting over 13,000 patients within the United States annually, it has a five-year survival rate of only 7% [2]. Primary treatments for GBM include maximal safe surgical resection, radiation, and chemotherapy [3]. While immunotherapies have shown significant efficacy in other cancers such as lung, melanoma and bladder, treatment of GBM tumors have struggled to show the same gains in immunotherapy efficacy over conventional treatments. These failures may be due to the protective nature of the blood–brain barrier and the heterogeneity and complexity of the GBM tumor microenvironment (TME), including the presence of tumor-associated macrophages (TAMs) [4].

Macrophages make up a significant fraction of the GBM TME, and they have been reported to make up as much as 20–40% of the tumor mass in glioblastomas [5,6,7]. To discuss these cells, however, there needs to be a distinction between the two main subtypes of phagocytic cells within the TME. The first macrophage lineage discussed stems from brain resident innate phagocytic cells, designated microglia-derived macrophages. These cells are generally considered to be derived from the Erythro-myeloid precursors from the yolk sac. Recent evidence has shown, however, that nearly 25% of microglia derive from peripheral mononuclear cells [8,9]. The second of these macrophage subtypes is the innate immune macrophages that are typically derived from peripheral blood mononuclear cells (PBMCs). These macrophages develop and function around the body, and they infiltrate into the tissues where they are needed. Importantly, they can infiltrate into the tumor parenchyma, where they interact with the cancer cells. The relative percentages of each lineage within GBM specifically are contested, with some studies identifying as much as 80% as PBMC-derived macrophages, while others show higher percentages of microglia derived within the GBM TME [10,11]. Both of these lineages of macrophages perform important immune monitoring and other functions. Both PBMC-derived and microglial-derived macrophages have a highly plastic phenotype, dynamically monitor the environment, and stimulate or inhibit the resulting immune response. Unfortunately, because they have similar functions and plasticity and a limited number of cell type specific markers, there is a significant challenge within the field to adequately distinguish between the two lineages, particularly within human patients [11,12]. Therefore, in much of the research surrounding macrophage function and genetic control, both lineages are generally studied as combined TAMs or glioma-associated macrophages. Thus, we will discuss the macrophages throughout this paper as their combination, without major distinction.

As part of the innate immune system, macrophages are known for their phagocytic capability and their interaction with the inflammatory response. Macrophages have varied functions, depending on their cellular state. They have been shown to act as both the gas and the brake pedal on the immune system, depending on the cytokines they interact with. Their ability to stimulate the immune system in response to threats makes them a strong contender for therapeutic modulation. However, in the GBM microenvironment, they tend to suppress the immune system by increasing T cell exhaustion and decreasing the ability of the immune system to target the tumor [13].

Non-coding RNAs (ncRNAs) have recently come to light as significant molecules of interest in molecular biology research. With the completion of the Human Genome and Telomere to Telomere Sequencing Projects, it is clear there are significant amounts of RNA found within the cell that do not code for proteins but function by other mechanisms [14,15,16]. Their roles can include utilizing their own three-dimensional structures to influence critical functions within the cell at all levels, such as changing transcriptional activation and regulation, protein translation and localization, and signaling across cells via exosomes [14,17,18]. ncRNAs are canonically broken into two major groups: small ncRNAs such as microRNAs (miRNAs, miRs), piwi-interacting small RNAs (piwiRNAs), small nuclear and nucleolar RNAs (snRNAs, snoRNAs) and long ncRNAs that are greater than 200 nucleotides, named long non-coding RNAs (lncRNAs) and circular RNAs (circRNAs) [19,20]. For this review, we will focus on two major classes of ncRNAs: miRNAs and lncRNAs. NcRNAs have been shown to be multifunctional, where many miRNAs tightly regulate numerous genes, and lncRNAs also have multiple functions, from epigenetic to translational roles within the cell, as exemplified by research on miR-138 and Metastasis-Associated Lung Adenocarcinoma Transcript 1 (MALAT1), respectively [21,22].

Many ncRNAs have been implicated in the progression of GBM, and others have been identified to impact the polarization of macrophages. Here, we provide a review of the current literature behind the connections between the polarization of macrophages, the progression of GBM, and how ncRNAs link them both to each other.

## 2. Analysis of Current Research

### 2.1. Macrophages in the Progression of GBM

Macrophages are a desirable target for the treatment of GBM. Preclinical studies have shown that macrophage modulation can halt GBM progression [23,24]. Macrophages in their naïve state are M0 but can be polarized into specific phenotypes such as M1 or M2 based on their activation state [11]. M1 macrophages are considered classically active and bear markers such as CD86 and inducible Nitric Oxide Synthase (iNOS); they are considered immunostimulatory [25]. These M1 macrophages are stimulated by interferon-γ (IFNγ), lipopolysaccharides, and other bacterial molecules [26]. M2 macrophages are considered to be alternatively activated; they bear identifying markers like CD163 and CD204 and are considered anti-inflammatory [27]. These macrophages are stimulated by cytokines such as IL-4, IL-10 and IL-13. M2 macrophages are more likely to release cytokines like IL-10 or chemokines like CCL17, which tend to lead to tumor progression [27]. Macrophages that have infiltrated the TME are then classified as TAMs. Once they are within the tumor, their classification becomes M1-like or M2-like based on expression of the markers above. GBM tumors specifically have a high infiltration of TAMs bearing M2-like markers, acting as anti-inflammatory modulators of the TME [28]. This leads to a decrease in the effectiveness of the immune system within the TME, as the anti-inflammatory mechanisms released by the M2-like TAMs suppress the immune system.

Within the TME, there are many mechanisms used by tumors to prevent immune stimulation. In GBM specifically, the immune system plays a complex role in tumor progression [29]. Within this microenvironment, the tumors release cytokines like vascular endothelial growth factor and Colony-Stimulating Factor 1, which recruit M2-like TAMs [30,31]. Once accumulated within the tumor, M2-like TAMs minimize the efficacy of effector T cells (T_eff_) through secreted factors like IL-10 and Transforming Growth Factor beta (TGF-β). The TAMs further recruit T regulatory cells (T_reg_) to the TME, blunting T_eff_ activation. Myeloid-derived suppressor cells (MDSCs) are also recruited to the TME, where they can further depress T_eff_ function, and in some cases can even be polarized into M2-like TAMs [32,33]. With all these immunosuppressive mechanisms, GBM tumors have proven resistant to immunotherapeutic modulation using conventional agents [4,34].

With the current success of immunotherapeutic interventions in other cancers, there remains interest in translating these successes to GBM. Currently, there are a few immunotherapy drugs that have been promising enough to proceed into phase III trials. These include the anti-PD-1 drugs Nivolumab [35,36] and Pembrolizumab [37,38], with anti-CTLA4 drugs such as Ipilimumab in early stage 1 and 2 trials as well [39,40]. However, these treatments alone have not shown significant extension of overall survival. Additionally, some trials using pembrolizumab have noted the impact of the immunosuppressive TME—and macrophages specifically—on the efficacy of treatments [41].

Chimeric Antigen Receptor T cell Therapy (CAR-T) therapy has been efficacious in blood cancers [42]. There is hope that CAR-T cell therapy will be effective in GBM, but the current CAR-T trials remain in the early phases of treatment [43,44]. Of interest, the presence of TAMs is a significant contributor to T cell exhaustion and yet does not have a current therapeutic modulation [45]. This does lend itself to an area of opportunity in terms of research and development. By modulating the polarization of the TAMs, there may be an opportunity to transform the GBM TME into a hostile survival environment for tumor cells.

### 2.2. ncRNAs in the Progression of GBM

There are a number of miRNAs and lncRNAs that have been identified to play a role in GBM, some as potential prognostic markers and others as potential therapeutic targets [46]. miRNAs are small RNAs, averaging 22 nucleotides (nt) in length, that are loaded into the RNA-induced silencing complex where they match up with messenger RNAs of genes that match with their 8 nt seed region. In this capacity, many miRNAs are able to target entire families of genes and have been known to regulate many critical cellular pathways [47,48]. This can be seen within the brain specifically through the actions of Lethal-7 family member a (Let-7a). Let-7a was one of the first miRNA identified, first in *C. elegans*, then in humans, and it has been shown to interact with genes like Beclin1 in microglia and cleaved poly ADP-ribose polymerase within the brain, controlling pathways such as autophagy and cell death and regulating the tight junctions within the blood–brain barrier (BBB) [49,50,51].

lncRNAs are a vast family of ncRNAs broadly defined as greater than 200 nt in length. They have a wide range of functions, interacting with all parts of cellular life and function, from genomic regulation all the way to post-translational regulation of genes [17]. Many lncRNAs have also been shown to have multiple different functions within the cell. For example, lncRNA HOX transcript Antisense RNA (HOTAIR) has been shown to control transcription downstream of the HoxD locus, while it has also been shown to regulate the Polycomb Repressive Complex 2 through direct protein interactions, and it can act as a competitive endogenous RNA, sequestering miRNAs like mir-130a [52,53,54]. In this capacity, HOTAIR has been shown to regulate more than one process within cells, and this is not unique to HOTAIR, as many identified lncRNAs have been shown to have multiple functions [55]. These tightly regulated nucleic acids show unique potential for therapeutic modulation, as their multiple functions allow therapeutics to target several pathways. This may then limit the ability of cancer cells to utilize redundant pathways to avoid cell death.

**Figure 1:**
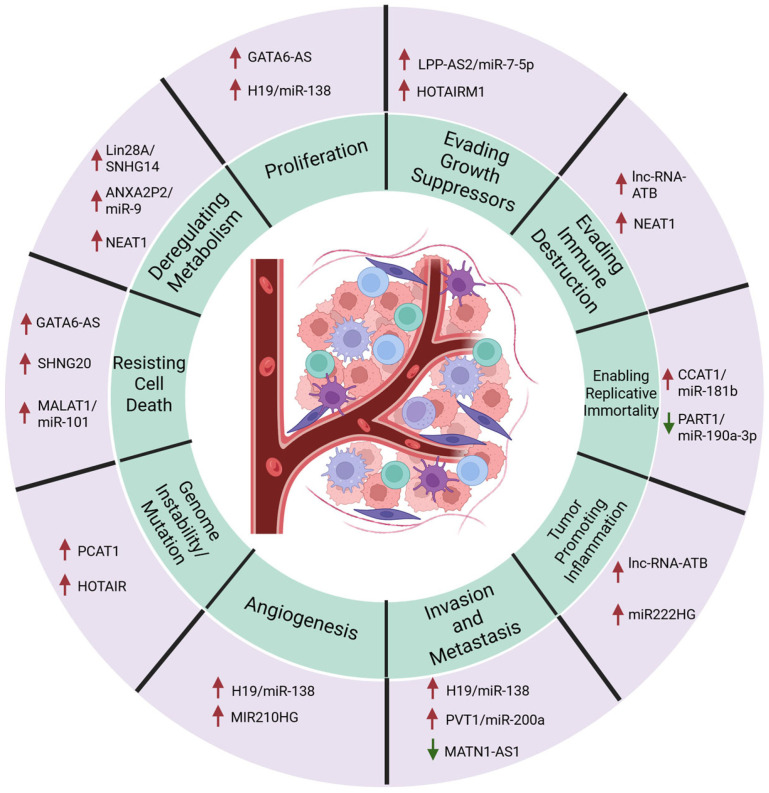
Hallmarks of cancer and the ncRNAs that control GBM progression. The green circle contains each of the hallmarks of cancer. The purple outer circle shows the ncRNAs that interact with pathways critical to each pathway. LncRNA/miRNA pairs indicate a miRNA sponging pathway: stated lncRNA binds to and blocks stated miRNA, preventing function. The arrows correspond to the effect each ncRNA has on the hallmark. A red up arrow indicates the presence of this ncRNA increases the progression of this hallmark within GBM. A green down arrow indicates the presence of the ncRNA decreases progression towards the hallmark [54,55,56,57,58,59,60,61,62,63,64,65,66,67,68,69,70,71]. Modified from Hanahan and Weinberg, 2011 [56]. Created in BioRender. Westemeier-Rice, E. (2025). https://BioRender.com/dlqs5fa.

Specifically, many ncRNAs identified in GBM have been identified as modulators of the hallmarks of cancer, with representative ncRNAs shown in Figure 1 [56,57,58,59,60,61,62,63,64,65,66,67,68,69,70,71,72,73,74]. Each of the hallmarks of cancer has shown at least one ncRNA modulating important pathways to impact progression, as defined by Hanahan and Weinberg in 2011 [56]. In particular, many of these ncRNAs impact more than one hallmark of cancer, such as H19, which increases proliferation and metastasis through mir138 sponging [58]. Neat1 has been shown to be stabilized by RNA-binding proteins, which allows for a more efficient activation of the Programmed Death Ligand 1 (PD-L1) pathway, helping to evade immune destruction while simultaneously impacting glycolysis [74,75]. lncRNA-ATB evades immune destruction by modulating the TME into a tumor-promoting inflammatory immune response [63]. This suggests a multifunctional role for many ncRNAs within GBM, which aligns with current research, as mentioned previously.

Of the most recently established hallmarks, the impact of ncRNAs on the ability to modulate the immune system and TME has attracted significant research attention, with most papers indexed by PubMed after the year 2019 [76]. However, within GBM specifically, there have been very few papers indexed since 2019 that directly correlate ncRNA’s impacts on macrophage polarization with GBM progression. Importantly, progress on understanding the immune system is crucial for progress across the body; however, the brain and central nervous system require additional specific research, not only because of the BBB but because of the presence of both the classical immune system and the CNS immune system of microglia and astrocytes. These two groups of immune cells have a complex interplay that needs to be accounted for. The discussion of ncRNAs below may shed some light on the complexity of the relationships between the immune system and the tumor itself.

### 2.3. ncRNAs and Their Roles in Macrophage Polarization

Macrophages are a critical part of immune function. As mentioned previously, their function is related to their phenotypic expression of markers, skewing towards either an M1 or an M2 phenotype. Because ncRNAs play a crucial role in the regulation of gene expression and cell function, it can be expected that there are ncRNAs that help regulate this skewing of phenotypic expression. There has been significant research into this, as researchers try to understand the complexity of the driving factors behind macrophage polarization and how these cells utilize phenotypic plasticity to influence and be influenced by their environment. Shown in Supplementary Table S1 is a representative sample of the ncRNAs that have been identified to modulate the polarization of macrophages into M1 or M2. Many of these ncRNAs are important complex regulators of polarization. Table 1 shows the ncRNAs specifically related to macrophage polarization within GBM. Both of these tables combined show a complex role for ncRNAs across disease states, with many of the ncRNAs implicated in GBM also playing an important role in other disease states, such as Neat1 [77,78] or miR21 [79,80,81].

### 2.4. Macrophage-Modulating ncRNAs Identified Within GBM

**Figure 2:**
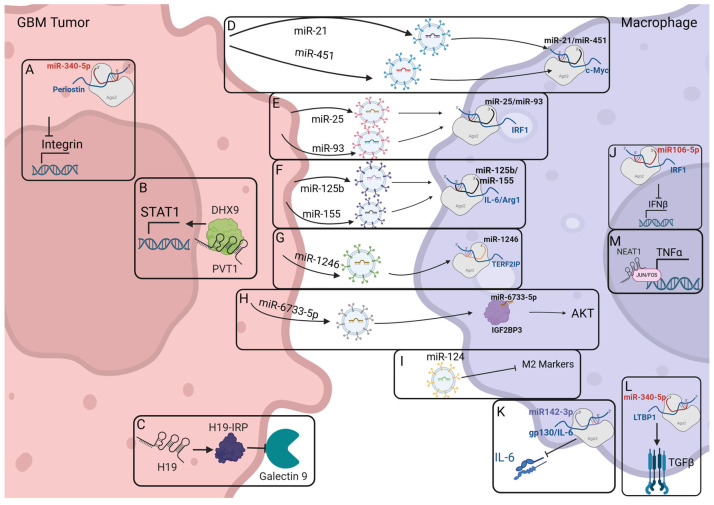
ncRNAs implicated in both GBM progression and macrophage polarization. (**A**). miR-340-5p regulates expression of Periostin to decrease integrin expression. (**B**). PVT1 stabilizes DHX9, increasing STAT1 signaling. (**C**). H19 encodes a small immune-related protein which decreases Galectin 9 Signaling. (**D**). miR-21 and miR-451 are packaged in exosomes and regulate expression of c-Myc. (**E**). miR-25 and miR-93 are packaged into exosomes and target IRF1 mRNA for degradation. (**F**). miR-125b and miR-155 are packaged into exosomes and traffic to macrophages to target IL-6 and Arginase 1. (**G**). miR-1246 is packaged into exosomes and interacts with the STAT3 signaling pathway to increase VEGF signaling. (**H**). miR-6733-5p travels to macrophages via exosomes, where it targets IGF2BP3. (**I**). miR-124 has been utilized in exosome research to decrease M2 markers within macrophages. (**J**). miR106-5p regulates expression of IRF1, decreasing expression of IFNß and related genes. (**K**). miR-142-3p targets the gp-130 subunit of IL-6, impairing IL-6 signaling in GBM. (**L**). miR-340-5p targets LTBP1 for degradation, increasing TGFβ signaling. (**M**). Neat1 stabilizes transcription factors Jun and Fos, increasing expression of TNFα. Created in BioRender. Westemeier-Rice, E. (2025) https://BioRender.com/54uu0p6.

Current research has identified few ncRNAs directly tied to macrophage polarization in GBM. As shown above, there are many ncRNAs directly tied to polarization pathways; however, very few of them have been directly implicated in GBM specifically. Those that have significant experimental evidence are shown in Figure 2. These include miR-21, miR-25, miR-93, miR-106-5p, miR-124, miR-125b, miR-142-3p, miR-155, miR-340-5p, miR-451, miR-1246, miR-6733-5p, H19, NEAT1, and PVT1 [78,81,82,83,84,85,86,87,88,89,90,91]. These ncRNAs play important roles in the crosstalk between the tumor and macrophages. Between cell-to-cell communication via exosomes and signaling interruptions, these ncRNAs function to increase the immune suppressive TME while promoting tumor growth and progression.

Some ncRNAs function within the GBM tumor cells to increase immunosuppressive cell signaling. For example, Liu et al. showed that miR-340-5p interacts with periostin in GBM cells to increase M2 TAM recruitment into the tumor parenchyma via integrin signaling (Figure 2A) [87]. Huang et al. showed that PVT1 functions within GBM cells to stabilize DExH-box helicase 9 (DHX9). This increases transcription of STAT1 and CX3CL1, leading to M2-like TAM recruitment (Figure 2B) [91]. Interestingly, Chen et al. identified a small protein originating from lncRNA H19 that binds and increases access to the promoters of CCL2 and Galectin-9, stimulating immunosuppressive TMA recruitment (Figure 2C) [90]. So far, significant research has focused on understanding the immunosuppressive signaling pathways and how ncRNAs may be targets to decrease immunosuppression via the tumor cells.

Many of the miRNAs that have shown direct evidence of macrophage modulation within GBM function through exosome packaging and delivery across cell types. Van der Vos et al. describes how miR-21 and miR-451 are packaged into exosomes originating from the GBM tumor cells. They were then taken up by the surrounding microglia and found to target the tumor suppressor c-Myc (Figure 2D) [81]. Work by Tankov et al. uncovered that miR-25 and mir-93 are packaged into extracellular vesicles by GBM cells under hypoxic conditions. They then showed that these hypoxic-derived exosomes loaded with miRNAs can impact macrophage polarization via decreasing cGAS pathway activity and decreasing type 1 IFN production (Figure 2E) [82]. Further, work by da Silva et al. discovered the presence of miR-125b and miR-155 in the secretome of GBM cells. Abrogating the presence of these miRNAs when co-incubated with macrophages led to an increase in IL-6 and Arginase-1 protein expression, increasing macrophage activation (Figure 2F) [85]. Qian et al. showed that under hypoxic conditions, miR-1246 is loaded into exosomes by glioma cells where it is delivered to TAMs. Once present within TAMs, miR-1246 targets telomeric repeat binding factor 2 interacting protein (TERF2IP), activating the STAT3 pathway (Figure 2G) [88]. Additionally, Huang et al. identified miR-6733-5p to be packaged into exosomes by glioma cells. These miRNA-loaded vesicles are then absorbed by macrophages, where the miRNA can target the RNA binding protein Insulin Growth Factor 2 Binding Protein 3 to stabilize the Akt pathway and increase M2 markers (Figure 2H) [89]. This has prompted additional research into utilizing exosomes as a packaging tool for delivery of miRNAs into GBM cells and macrophages. Work on miR-124 by Hong et al. shows that delivery of miR-124 via exosomes effectively abrogates GBM growth and decreases expression of M2 markers in macrophages (Figure 2I) [84]. These miRNA-loaded exosomes are commonly generated by GBM tumors and then trafficked to the macrophages, where they impact polarization.

The final mechanism of ncRNA modulation of macrophage polarization we will cover is from within the macrophages themselves. As described by Shi et al., miR-106-5p acts on the mRNA for IRF1. This decreases IRF1 gene expression and downstream progression of the IFN-β signaling cascade, leading to an increase in M2 polarization within the macrophages (Figure 2J) [83]. Additional research by Sonda et al. on miR-142-3p downregulation in GBM identified an interaction between miR-142-3p and the 3′ untranslated region of the gp130 subunit of IL-6 in macrophages. This significantly decreased IL-6 expression. However, because miR-142-3p is downregulated in GBM, the corresponding increase in IL-6 leads to a more immune-suppressive phenotype for the macrophages (Figure 2K) [86]. Liu et al. also discovered miR-340-5p functions within macrophages by targeting latent transforming growth factor beta binding protein 1 (LTBP1) to increase M2 polarization (Figure 2L) [87]. Characterized by Toker et al., Neat1 is highly expressed in GBM and macrophages, where it impacts expression of transcription factors like Jun and Fos, increasing inflammation and myeloid infiltration and function (Figure 2M) [78]. Modulating the macrophage polarization via ncRNAs within the macrophages presents a unique opportunity to bypass the GBM-enforced signals and identify new immunostimulatory pathways.

Interestingly, many of these specific ncRNAs have more than one function within the cell, and many have been found to have roles beyond only macrophage polarization. Mir-21 has been shown to influence M1 and M2 polarization across different tissue types, but as one of the first mammalian miRNAs discovered, it has been classified in many different types of cancers and has been shown to be both pro and anti-tumor depending on the context [92,93,94,95,96]. PVT1 has also been shown to modulate macrophage polarization, but it also impacts processes within the GBM tumor itself, increasing the invasive capability of the tumor [65,91]. These ncRNAs could be used as important prognostic markers, helping to identify if patients might benefit from immunotherapies or if macrophage-modulating treatments might better impact treatment efficacy.

Overall, it can be concluded that the combined overarching regulation provided by the ncRNAs in GBM and in macrophages contributes to a highly immunosuppressive TME. There is constant communication between the cell types via miRNA and lncRNA loaded exosomes; this provides a bridge utilized by the tumor cells to inhibit macrophage anti-tumor function and increase the presence of immunosuppressive TAMs.

### 2.5. Connections Between ncRNAs in Macrophages and How They Impacts GBM Progression

The crosstalk between macrophages and GBM tumor cells is highly dynamic, with recent technological advancements like single-cell RNA sequencing and spatial transcriptomics identifying the heterogeneity and plasticity of the macrophages within the TME [97,98]. Importantly, this crosstalk expands beyond the classical direct cell-to-cell contact or cytokine release and is now known to extend to ncRNA-loaded exosomes. These ncRNAs are able to send polarization signals, further driving the macrophages into pro- or anti-tumorigenic states. Many dysregulated ncRNAs in GBM lead to a more pro-tumorigenic state, but there are some that offer an anti-tumorigenic M1-like polarization for the macrophages, such as Neat1 [78].

ncRNAs strongly influence the polarization of macrophages. There has been significant debate regarding the pro- or anti-inflammatory nature of many of these pathways in relation to their effects on macrophages. Further studies on the impacts of the field of ncRNAs may elucidate some of these conflicting studies, characterizing their role in promoting or inhibiting tumor progression. Within the GBM environment, macrophages are highly likely to polarize into M2-like phenotypes [99]. While this has conventionally been attributed to the presence of cytokines and other protein signals, current research has shown the impact of ncRNAs cannot be ignored [27,100]. These ncRNAs add another layer of regulation, additional research on which may be useful to promote the anti-tumor M1-like polarization, leading to increased anti-tumor efficacy and immune stimulation.

### 2.6. Clinical Relevance

There is currently an unmet need to better understand the barriers to immunotherapy treatments in GBM. Because immunotherapy has shown limited efficacy within GBM, preclinical evidence has shown immune modulators, such as macrophages, are promising targets for creating immunologically active TMEs [101,102]. With a better understanding of how ncRNAs impact the polarization and activation of macrophages, we can identify new avenues of therapeutic intervention and combination therapies to better prevent the spread of GBM.

Currently, according to the United States’ National Institutes of Health Clinical Trial Monitoring system (Cliniticaltrials.gov) and using keyword searches of “Glioblastoma Multiforme”, “miRNA” and “lncRNA”, there are only four studies, of which three are actively recruiting, that are studying the effects of ncRNAs as diagnostic markers but not as current targets for therapeutics [103]. There has been one phase zero study within recurrent GBM utilizing the RNA interference pathway [104]. In this trial, researchers utilized the function of small interfering RNAs (siRNAs) targeting the Bcl2Like12 gene, an important GBM oncogene. This siRNA was loaded into spherical nucleic acids that were designed to penetrate the brain. This phase 0 trial saw limited toxicity and effective uptake of the siRNA, with a resulting decrease in Bcl2Like12 expression [104]. This is promising for the progression of RNA-based therapeutics, with the question of brain penetration being a significant barrier. There is more work to be done to further this research, but the promise of minimal toxicity and effective gene targeting is an exciting development. This offers the opportunity to utilize a similar process to use siRNAs to target lncRNAs like H19 or PVT1, which play important roles in the progression of GBM [90,91]. Further, this technology could be used to deliver antago-miRs, which are small pieces of RNAs designed to interact with miRNAs and block their function. This could be useful, for example, for targeting miR-142-3p, which generally blocks translation of IL-6 subunit gp130 [86]. This could offer the chance to increase immune response to the tumor by upregulating the expression of IL-6 on macrophages. Recent clinical trials outside of GBM have shown minor efficacy across diseases such as cystic fibrosis, lung cancer, and mesothelioma; however, none have moved beyond Emergency FDA approval, and none have been moved to market [105,106,107,108]. These still represent significant and exciting progress towards an ncRNA-based therapeutic in the future.

Modulating the ncRNA transcriptome offers a novel perspective on cancer treatment. Current therapeutic interventions in clinical trials are primarily multi-treatment-based, such as combination therapies of nivolumab and Ipilimumab [40] or pembrolizumab and bevacizumab [38]. Unfortunately, in both trials, the monotherapy of nivolumab or pembrolizumab worked better than the combination. However, there are still opportunities to test these with ncRNA-modulating therapies, such as antago-miRs or siRNAs, to enhance targeting or minimize the crosstalk between the tumor and the macrophages, allowing for the immunotherapy to function more effectively.

## 3. Discussion and Future Avenues of Research

Herein, we have discussed the roles of many miRNAs and lncRNAs that play a role in the polarization and localization of macrophages to the GBM TME. We have discussed many of the most prevalent ncRNAs and their roles impacting the hallmarks of cancer within GBM. We have also identified many of the ncRNAs that play an important role in macrophage polarization across a multitude of disease states, from cancer to spinal cord injuries to healthy monocytes. We have also discussed the overlap between the ncRNAs identified to play a role in GBM progression and macrophage polarization, identifying the impact of these ncRNAs on prognosis related to the TME.

As the macrophage populations within the GBM TME can be monocyte-derived or microglia-derived in varying proportions, it is important to note that most of the research presented here does not specify how this lineage impacts ncRNA expression or function. Because microglia and infiltrating macrophages are highly similar in markers and function within the TME, there is significant debate among neuroimmunologists about perfectly isolating cell markers [109,110]. Thus, there is a discrepancy between what ncRNAs can be attributed to each lineage specifically. Therefore, there is a critical need for research to differentiate between the ncRNA landscape of microglia- and monocyte-derived macrophages. However, this will likely only be possible once the success of single-cell sequencing methods and other individualized transcriptomic profiling methods allows for the specific isolation of microglia-derived TAMs from monocyte-derived TAMs.

While not strongly focused on here, there is additional research being carried out to understand the roles of circRNAs in macrophage polarization and GBM progression [111,112]. In particular, circRNAs are specifically abundant within the brain, as compared to other organ systems [113]. They have been strongly linked to neuronal development, aging, and neurodegenerative diseases. This presents an intriguing path of research into how these circular RNAs impact not only brain function but the recruitment of macrophages and microglia to the TME.

With much of the research in the ncRNA field, the tissue location of interest must be accounted for during the research. ncRNAs are highly tissue-specific, and their functions relate to their locations [114,115]. This leads to discrepancies within research regarding many ncRNAs. Their functions may be highly anti-inflammatory in some tissues but pro-inflammatory in others. Because of this, there needs to be increased research into these ncRNAs over a broad spectrum of tissues and disease states. This will allow for more targeted therapeutics, utilizing personalized medicine to wrangle the immune system to more effectively target diseases such as cancer. Recent reviews have discussed this plasticity in depth, agreeing with the need for additional research on the different ncRNAs to understand their tissue-dependent impacts on macrophage polarization [116,117,118].

The study of the immune system is the study of balance. Cancer occurs when that balance is disrupted and the immune system is unable to respond correctly to the rapidly dividing cells. As demonstrated here, the polarization of macrophages and TAMs is tightly controlled not only at the translational and post-translational level but at the transcriptional level as well. Many ncRNAs work as a high-level control, ensuring that the levels of mRNAs remain within established bounds. When pulled out of balance, whether it be through packaging in exosomes or external conventional signaling mechanisms, ncRNAs may be the key to therapeutically targeting these critical pathways to ensure that the immune system remains in balance and fights tumors more effectively.

## Figures and Tables

**Table 1:** NcRNAs impacting macrophage polarization within GBM.

ncRNA Acronym	Full Name	Polarization	Targets/Function	Citation
Mir21		Increases M2 Polarization	Loaded into exosomes, targets c-Myc in microglia	van der Vos K, et al. [81]
Mir25		Decreases M1 Polarization	Mir25 and mir93 are loaded into exosomes, where they can target the cGAS-STING pathway in macrophages	Tankov S, et al. [82]
Mir93		Decreases M1 Polarization	Mir93 and mir25 are loaded into exosomes, where they can target the cGAS-STING pathway in macrophages	Tankov S, et al. [82]
mir106-5p		Increases M2 Polarization	Targets the IFNβ pathway, inhibiting IRF1 signaling	Shi Y, et al. [83]
miR124		Decreases M2 Polarization	Loaded into exosomes and decreases STAT3 signaling, preventing M2 recruitment and tumor progression	Hong S, et al. [84]
Mir125b		Increases M2 Polarization	Targets IL-6 and Arginase-1, decreasing immune stimulation	Da Silva KC, et al. [85]
mir142-3p		Decreases Macrophage Polarization	blocks translation of gp130 subunit of IL-6	Sonda N, et al. [86]
mir155		Increases M2 Polarization	Targets IL-6 and Arginase-1, decreasing immune stimulation	Da Silva KC, et al. [85]
Mir340-5p		Decreases M2 Polarization	Through its interactions with Periostin, it prevents M2-like TAM recruitment to the TME	Liu Y, et al. [87]
mir451		Increase M2 Polarization	Loaded into Exosomes, targets c-Myc in microglia	van der Vos K, et al. [81]
mir1246		Increases M2 Polarization	Loaded into exosomes and delivered to macrophages, targeting TERF2IP, activating the STAT3 Pathway	Qian M, et al. [88]
miR6733-5p		Increase M2 Polarization	Loaded into exosomes and delivered to macrophages, targeting the AKT pathway via IGF2BP3	Huang S, et al. [89]
H19		Increases M2 Polarization	Encodes a small immune protein, H19-IRP, which prevents transcription of CCL2 and Galectin-9	Chen J, et al. [90]
NEAT1	Nuclear Paraspeckle Assembly Transcript 1	Increases M1 Polarization	Enriches TNFα and NF-κB pathways and downstream genes while decreasing inflammatory cytokine expression	Toker J, et al. [78]
PVT1	Plasmacytoma variant translocation 1	Increases M2 Polarization	Stabilizes DHX9, which acts as a transcription factor for STAT1 and CX3CL1	Huang L, et al. [91]

## Data Availability

No new data were created or analyzed in this study. Data sharing is not applicable to this article.

## Supplementary Materials

The following supporting information can be downloaded at: https://www.mdpi.com/article/10.3390/cells14191528/s1. Table S1: NcRNAs impacting Macrophage Polarization across disease states. Refs. [77,78,79,81,82,83,84,85,86,87,88,89,90,91,92,93,119,120,121,122,123,124,125,126,127,128,129,130,131,132,133,134,135,136,137,138,139,140,141,142,143,144,145,146,147,148,149,150,151,152] are cited in Supplementary Materials.

## Abbreviations

The following abbreviations are used in this manuscript:

GBM	Glioblastoma multiforme
TME	Tumor microenvironment
RNA	Ribonucleic acid
ncRNA	Non-coding RNA
miRNA	MicroRNA
miR	MicroRNA
piwiRNA	Piwi-interacting RNA
snRNA	Small nuclear RNA
snoRNA	Small nucleolar RNA
lncRNA	Long non-coding RNA
lincRNA	Long intergenic non-coding RNA
circRNAs	Circular RNA
MALAT1	Metastasis-associated lung adenocarcinoma transcript 1
iNOS	Inducible nitric oxide synthase
IFNγ	Interferon—gamma
TAMs	Tumor-associated macrophages
T_eff_	Effector T cells
TGF-β	Transforming growth factor beta
T_reg_	T regulatory cells
MDSCs	Myeloid-derived suppressor cells
CAR-T	Chimeric antigen receptor T cell therapy
nt	Nucleotides
Let7a	Lethal-7 family microRNA A
BBB	Blood–brain barrier
HOTAIR	HOX transcript antisense RNA
PD-L1	Programed cell death ligand 1
IL-6	Interleukin 6
Let7b	Lethal-7 family microRNA B
lncRNA ANCR	Antisense non-coding RNA in the INK4 locus
CCAT1	Colon cancer-associated transcript 1
Cox2	Cyclooxygenase 2
GAS5	Growth arrest-specific transcript 5
GNAS-AS1	GNAS-antisense transcript 1
Linc00662	Long intergenic non-coding RNA 00662
lincRNA p21	Long intergenic non-coding RNA p21
lncRNA RP11-361F15.2	Long non-coding RNA RP11-361F15.2
MEG3	Maternally expressed gene 3
lncRNA-MM2P	Long non-coding RNA macrophage m2 polarization
NIFK-AS1	NIFK-antisense 1
PACERR	P50-associated cyclooxygenase-2 extragenic RNA
PVT1	Plasmacytoma variant translocation 1
RPPH1	RNA component of ribonuclease P
TUC339	Transcribed non-coding RNA from ultra conserved element 339
XIST	X-inactive-specific transcript
DHX9	Dexh-box helicase 9
TERF2IP	Telomeric repeat binding factor 2 interacting protein
LTBP1	Latent transforming growth factor beta binding protein 1
siRNA	Small interfering RNA
