# Supplementary material for: Macrophages in Glioblastoma and How Non-Coding RNAs Impact Their Differentiation
Source: Cells. 2025 Sep 30;14(19):1528. doi: 10.3390/cells14191528 (PMC12523494; doi:10.3390/cells14191528)
Supplement: Supplementary file 1 [file cells-14-01528-s001.zip › cells-3874042-supplementary.pdf]

cells

MDPI

Supplemental Table S1: NcRNAs impacting Macrophage Polarization across disease states.

ncRNA Acronym	Full name	Cancer/Disease	Polarization	Targets/Function	Citation
mir16		Breast	decreases M2 polarization	decreases expression of IL-6 and TGF-β after treatment with anti-tumor epigallocatechin gallate	Jang, JY et al[119]
mir19a-3p		Breast	Increases M2 Polarization	downregulation increases expression of Fra-1, which increases invasion and metastasis	Yang J, et al [120]
Mir-21		Primary Human Monocyte	Increases M1 Polarization	packaged into exosomes, and when delivered to macrophages, increases pro-inflammatory cytokine release	Madhyastha R, et al[121]
		Primary Human Monocyte	Increases M1 Polarization	interaction with prostaglandin E2 prevents mir-21 from inhibiting M2 genes/markers	Wang Z, et al[122]
		Glioma	Increase M2 Polarization	Loaded into Exosomes, targets c-Myc in microglia	van der Vos K, et al[123]
mir24		Primary Human Monocyte	Increases M2 Polarization	inhibits cytokine release from LPS stimulation	Fordham J, et al[124]
Mir25		Glioma	Decreases M1 Polarization	Mir25 and mir93 are loaded into exosomes, where they can target the cGAS-STING pathway in macrophages	Tankov S, et al[125]
Mir29a-3p		Oral Squamous Cell	Increases M2 Polarization	packaged into exosomes, and when delivered to macrophages, decreased expression of Suppressor of Cytokine Signaling 1 and phosphorylation of STAT6	Cai J and Qiao B, et al[126]
Mir34a		Multiple Myeloma	Decreases M1 Polarization	targets TLR9, decreasing expression and impairing function	Zhang R, et al[127]
mir92a		Colorectal	Increases M2 Polarization	packaged into exosomes, where it activates MAPK/ERK pathway within macrophages	Zhao W, et al[128]
Mir93		Glioma	Decreases M1 Polarization	Mir93 and mir25 are loaded into exosomes, where they can target the cGAS-STING pathway in macrophages	Tankov S, et al[125]
mir106-5p		Glioma	Increases M2 Polarization	Targets IFNβ pathway, inhibiting IRF1 signaling	Shi Y, et al[129]
miR124		Glioma	Decreases M2 Polarization	Loaded into exosomes, and decreases STAT3 signaling, preventing M2 recruitment and tumor progression	Hong S, et al[130]
mir125a-5p		Primary Human Monocyte	Increases M2 Polarization	targets KLF13, decreasing T cell activation	Banerjee S, et al[131]
Mir125b		Glioma	Increases M2 Polarization	Targets IL-6 and Arginase-1, decreasing immune stimulation	Da Silva KC, et al[132]
mir127		Lung Inflammation	Increases M1 Polarization	binds to BCL2, which activates JNK kinase, leading to M1 macrophage polarization	Ying H, et al[133]
mir142-3p		Glioma	Decreases Macrophage Polarization	blocks translation of gp130 subunit of IL-6	Sonda N, et al[134]
mir145		Leukemia	Increases M2 Polarization	targets IL-16 and IL-10 mRNAs for degradation	Huang Y, et al[135]
mir146-a		Diabetes	Decreases M2 Polarization	targets TLR4 for degradation, decreasing NF-κB function	Peng X, et al[136]
mir155		Acute Lung Injury	Increases M1 Polarization	targets MSK1 for degradation, increasing activity of the p38/MAPK pathway	Xu Y, et al[137]
		Glioma	Increases M2 Polarization	Targets IL-6 and Arginase-1, decreasing immune stimulation	Da Silva KC, et al[132]
mir195-5p		Atherosclerosis	Decreases M1 Polarization	targets IL-1β, IL-6 and TNFα, while decreasing the phosphorylation of JNK and MAPK	Bras JP, et al[138]
mir224		Spinal Cord Injury	Increases M2 Polarization	Is sponged by NEAT1 to allow expression of IL-33, thereby decreasing astrocyte activation	Liu D, et al[139]
mir301a-3p		Endometriosis	Increases M2 Polarization	Loaded into Exosomes and when taken in by macrophages, upregulates Arginase1, PTEN and PI3K	Huang Y, et al[140]
Mir340-5p		Glioma	Decreases M2 Polarization	Through its interactions with Periostin, it prevents M2-like TAM recruitment to the TME	Liu Y, et al[141]
mir451		Glioma	Increase M2 Polarization	Loaded into Exosomes, targets c-Myc in microglia	van der Vos K, et al[123]
mir511		Allergic Inflammation	Increase M2 Polarization	Increases expression of Arginase1 and decreases iNOS	Do D, et al[142]
mir720		Breast	decreases M2 polarization	Targets GATA3 mRNA	Zhong Y, et al[143]
mir1246		Glioma	Increases M2 Polarization	Loaded into exosomes and delivered to macrophages, targeting TERF2IP, which activates the STAT3 Pathway	Qian M, et al[144]
miR6733-5p		Glioma	Increase M2 Polarization	Loaded into exosomes and delivered to macrophages, targeting the AKT pathway via IGF2BP3	Huang S, et al[145]
let7a		Intracerebral hemorrhage	Increases M1 Polarization	After damage, let7a is decreased, allowing target protein CKIP-1 to increase proinflammatory cytokines	Yang Z, et al[146]
let7b		Prostate	Increases M2 Polarization	Decreases expression of IL-10, IL12, and IL23, while increasing expression of TNFα	Wang Z, et al[147]
lncRNA ANCR	antisense non-coding RNA in the INK4 locus	Gastric	Decreases M1 Polarization	targets FoxO1 for ubiquitin targeted degradation, decreasing expression of IL-1β and IL-6	Xie C, et al[148
CCAT1	Colon Cancer Associated Transcript 1	Prostate	Increases M2 Polarization	CCAT1 acts as a sponge for mir148a, which impacts expression of PKCζ	Liu J, et al[149]
Cox2	Cyclooxygenase 2	hepatocellular carcinoma	Decreases M1 Polarization Increases M2 Polarization	decreases expression of IL-12, iNOS and TNF-α in M1 macrophages, while increases expression of IL-10, Arg-1, and Fizz-1 in M2 macrophages	Ye Y et al[150]

GAS5	Growth Arrest Specific transcript 5	Multiple Sclerosis	Decreases M2 Polarization	Inhibits PRC2 from acting as a transcription factor for IRF4, by binding PRC2	Sun D, et al[151]
GNAS-AS1	GNAS-Antisense Transcript 1	Non-small cell lung cancer	Increases M2 Polarization	acts as a sponge for mir4319, which allows for expression of N-terminal EF Hand calcium binding protein 3	Li Z, et al[152]
H19		Glioma	Increases M2 Polarization	Encodes a small immune protein, H19-IRP. Which prevents transcription of CCL2 and Galectin-9	Chen J, et al[153]
HOTAIR	HOX transcript Antisense RNA	laryngeal squamous cell	Increases M2 Polarization	gets loaded into exosomes, and when introduced to the macrophages, interacts with PI3K to increase M2 polarization	Wang J, et al[154]
Linc00662		hepatocellular carcinoma	Increases M2 Polarization	increases expression of WNT3A, increasing signaling via WNT/β-catenin pathway	Tian X, et al[155]
lincRNA p21		Breast	Decreases M1 Polarization	Degrades p53 via MDM2, which lowers activation of NF-κB/STAT3	Zhou L, et al[156]
lncRNA RP11-361F15.2		Osteosarcoma	Increases M2 Polarization	sponges mir30c-5p, which allows for increased expression of cytoplasmic polyadenylation element binding protein 4	Yang D, et al[157]
MALAT1	metastasis associated lung adenocarcinoma transcript 1	Primary Human Monocyte	Increases M1 Polarization	acts as a sponge for mir30b, allowing for cytokine secretion	Ahmad I, et al[158]
MEG3	Maternally expressed gene 3	hepatocellular carcinoma	Increases M1 Polarization	Binds to human antigen R, which allows for CCL5 transcription	Wei H, et al[159]
lncRNA-MM2P	Long non-coding RNA macrophage m2 polarization	Primary Human Monocyte	Increases M2 Polarization	Prevents Phosphorylation of STAT6, continuing STAT6 activation	Cao J, et al[160]
NEAT1	Nuclear Paraspeckle Assembly Transcript 1	Spinal Cord Injury	Increases M2 Polarization	Sponges mir-224 to increase IL-33 expression, decreasing M2 Macrophage mediated astrocyte activation	Liu D, et al[139]
		Glioma	Increases M1 Polarization	Enriches TNFα and NF-κB pathways, and downstream genes, while decreasing inflammatory cytokine expression	Toker J, et al[161]
NIFK-AS1	NIFK-antisense 1	Endometrial carcinoma	Decreases M2 Polarization	acts as a sponge for mir-146a, which increases Notch expression	Zhou Y, et al[162
PACERR	p50-associated cyclooxygenase-2 extragenic RNA	Pancreatic ductal Carcinoma	Increases M2 Polarization	acts as a sponge for mir-671-3p, which allows for KLF12 to activate the p-AKT/c-Myc pathway. It also stabilizes KLF12 through interactions with IGF2BP2	Liu Y, et al[163]
PVT1	plasmacytoma variant translocation 1	Glioma	Increases M2 Polarization	stabilizes DHX9, which acts as a transcription factor for STAT1 and CX3CL1	Huang L, et al[164]
RPPH1	RNA component of ribonuclease P	Colorectal	Increases M2 Polarization	packaged into exosomes and when absorbed by macrophages, binds β-III tubulin, to prevent macrophage migration	Liang Z, et al[165]
TUC339	Transcribed non-coding RNA from ultra conserved element 339	hepatocellular carcinoma	Increases M2 Polarization	packaged into exosomes, and acts as a mediator from HCC cells to macrophages, decreasing immunostimulatory cytokine release	Li X, et al[166]
XIST	X-inactive-specific transcript	Burn Injuries	Increases M2 Polarization	acts as a sponge for mir-19b, increasing expression of IL-33	Pi L, et al[167]
		Spinal Cord Injury	Increases M2 Polarization	acts as a sponge for mir-124-3p, thereby increasing expression of IRF1	Yang J, et al[168]

Supplementary Citations

119. Jang, J.-Y.; Lee, J.-K.; Jeon, Y.-K.; Kim, C.-W. Exosome Derived from Epigallocatechin Gallate Treated Breast Cancer Cells Suppresses Tumor Growth by Inhibiting Tumor-Associated Macrophage Infiltration and M2 Polarization. *BMC Cancer* **2013**, *13*, 1–12, doi:10.1186/1471-2407-13-421.

120. Yang, J.; Zhang, Z.; Chen, C.; Liu, Y.; Si, Q.; Chuang, T.-H.; Li, N.; Gomez-Cabrero, A.; Reisfeld, R.A.; Xiang, R.; et al. MicroRNA-19a-3p Inhibits Breast Cancer Progression and Metastasis by Inducing Macrophage Polarization through Downregulated Expression of Fra-1 Proto. *Oncogene* **2014**, *33*, 3014–3023, doi:10.1038/onc.2013.258.

121. Madhyastha, R.; Madhyastha, H.; Nurrahmah, Q.I.; Purbasari, B.; Maruyama, M.; Nakajima, Y. MicroRNA 21 Elicits a Pro-Inflammatory Response in Macrophages, with Exosomes Functioning as Delivery Vehicles. *Inflammation* **2021**, *44*, 1274–1287, doi:10.1007/s10753-021-01415-0.

122. Wang, Z.; Brandt, S.; Medeiros, A.; Wang, S.; Wu, H.; Dent, A.; Serezani, C.H. MicroRNA 21 Is a Homeostatic Regulator of Macrophage Polarization and Prevents Prostaglandin E2-Mediated M2 Generation. *PLOS ONE* **2015**, *10*, e0115855, doi:10.1371/journal.pone.0115855.

123. Van Der Vos, K.E.; Abels, E.R.; Zhang, X.; Lai, C.; Carrizosa, E.; Oakley, D.; Prabhakar, S.; Mardini, O.; Crommentuijn, M.H.W.; Skog, J.; et al. Directly Visualized Glioblastoma-Derived Extracellular Vesicles Transfer RNA to Microglia/Macrophages in the Brain. *Neuro-Oncol.* **2016**, *18*, 58–69, doi:10.1093/neuonc/nov244.

124. Fordham, J.B.; Naqvi, A.R.; Nares, S. miR-24 Regulates Macrophage Polarization and Plasticity. *J. Clin. Cell. Immunol.* **2015**, *06*, doi:10.4172/2155-9899.1000362.
125. Tankov, S.; Petrovic, M.; Lecoultre, M.; Espinoza, F.; El-Harane, N.; Bes, V.; Chliate, S.; Bedoya, D.M.; Jordan, O.; Borchard, G.; et al. Hypoxic Glioblastoma-Cell-Derived Extracellular Vesicles Impair cGAS-STING Activity in Macrophages. *Cell Commun. Signal.* **2024**, *22*, 1–21, doi:10.1186/s12964-024-01523-y.
126. Cai, J.; Qiao, B.; Gao, N.; Lin, N.; He, W. Oral Squamous Cell Carcinoma-Derived Exosomes Promote M2 Subtype Macrophage Polarization Mediated by Exosome-Enclosed miR-29a-3p. *Am. J. Physiol.-Cell Physiol.* **2019**, *316*, C731–C740, doi:10.1152/ajpcell.00366.2018.
127. Zhang, R.; Zhang, D.; Luo, Y.; Sun, Y.; Duan, C.; Yang, J.; Wei, J.; Li, X.; Lu, Y.; Lai, X. miR-34a Promotes the Immunosuppressive Function of Multiple Myeloma-Associated Macrophages by Dampening the TLR-9 Signaling. *Cancer Med.* **2024**, *13*, e7387, doi:10.1002/cam4.7387.
128. Zhao, W.; Wu, Y.; Wang, Y.; Li, T.; Liu, Q.; Hou, Z. Exosomal miR-92a-3p Modulates M2 Macrophage Polarization in Colorectal Cancer: Implications for Tumor Migration and Angiogenesis. *Med. Oncol.* **2025**, *42*, 1–15, doi:10.1007/s12032-025-02635-2.
129. Shi, Y.; Zhang, B.; Zhu, J.; Huang, W.; Han, B.; Wang, Q.; Qi, C.; Wang, M.; Liu, F. miR-106b-5p Inhibits IRF1/IFN-β Signaling to Promote M2 Macrophage Polarization of Glioblastoma. *OncoTargets Ther.* **2020**, *13*, 7479–7492, doi:10.2147/OTT.S238975.
130. Hong, S.; You, J.Y.; Paek, K.; Park, J.; Kang, S.J.; Han, E.H.; Choi, N.; Chung, S.; Rhee, W.J.; Kim, J.A. Inhibition of Tumor Progression and M2 Microglial Polarization by Extracellular Vesicle-Mediated microRNA-124 in a 3D Microfluidic Glioblastoma Microenvironment. *Theranostics* **2021**, *11*, 9687–9704, doi:10.7150/thno.60851.
131. Banerjee, S.; Cui, H.; Xie, N.; Tan, Z.; Yang, S.; Icyuz, M.; Thannickal, V.J.; Abraham, E.; Liu, G. miR-125a-5p Regulates Differential Activation of Macrophages and Inflammation*. *J. Biol. Chem.* **2013**, *288*, 35428–35436, doi:10.1074/jbc.M112.426866.
132. da Silva, K.C.; Lima, I.S.; Santos, C.C. dos; Nonaka, C.K.V.; Souza, B.S. de F.; David, J.M.; Ulrich, H.; Nascimento, R.P. do; Costa, M. de F.D.; dos Santos, B.L.; et al. Agathisflavone Inhibits Viability and Modulates the Expression of miR-125b, miR-155, IL-6, and Arginase in Glioblastoma Cells and Microglia/Macrophage Activation. *Molecules* **2025**, *30*, 158, doi:10.3390/molecules30010158.
133. Ying, H.; Kang, Y.; Zhang, H.; Zhao, D.; Xia, J.; Lu, Z.; Wang, H.; Xu, F.; Shi, L. MiR-127 Modulates Macrophage Polarization and Promotes Lung Inflammation and Injury by Activating the JNK Pathway. *J. Immunol.* **2015**, *194*, 1239–1251, doi:10.4049/jimmunol.1402088.
134. Sonda, N.; Simonato, F.; Peranzoni, E.; Calì, B.; Bortoluzzi, S.; Bisognin, A.; Wang, E.; Marincola, F.M.; Naldini, L.; Gentner, B.; et al. miR-142-3p Prevents Macrophage Differentiation during Cancer-Induced Myelopoiesis. *Immunity* **2013**, *38*, 1236–1249, doi:10.1016/j.immuni.2013.06.004.
135. Huang, Y.; Du, K.L.; Guo, P.Y.; Zhao, R.M.; Wang, B.; Zhao, X.L.; Zhang, C.Q. IL-16 Regulates Macrophage Polarization as a Target Gene of Mir-145-3p. *Mol. Immunol.* **2019**, *107*, 1–9, doi:10.1016/j.molimm.2018.12.027.
136. Peng, X.; He, F.; Mao, Y.; Lin, Y.; Fang, J.; Chen, Y.; Sun, Z.; Zhuo, Y.; Jiang, J. miR-146a Promotes M2 Macrophage Polarization and Accelerates Diabetic Wound Healing by Inhibiting the TLR4/NF-κB Axis. *J. Mol. Endocrinol.* **2022**, *69*, 315–327, doi:10.1530/JME-21-0019.
137. Xu, Y.; Zhang, C.; Cai, D.; Zhu, R.; Cao, Y. Exosomal miR-155-5p Drives Widespread Macrophage M1 Polarization in Hypervirulent Klebsiella Pneumoniae-Induced Acute Lung Injury via the MSK1/P38-MAPK Axis. *Cell. Mol. Biol. Lett.* **2023**, *28*, 92, doi:10.1186/s11658-023-00505-1.
138. Bras, J.P.; Silva, A.M.; Calin, G.A.; Barbosa, M.A.; Santos, S.G.; Almeida, M.I. miR-195 Inhibits Macrophages pro-Inflammatory Profile and Impacts the Crosstalk with Smooth Muscle Cells. *PLoS ONE* **2017**, *12*, e0188530, doi:10.1371/journal.pone.0188530.

139. Liu, D.; Wei, Y.; Liu, Y.; Wu, T.; Hu, J.; Lu, H. The Long Non-Coding RNA NEAT1/miR-224-5p/IL-33 Axis Modulates Macrophage M2a Polarization and A1 Astrocyte Activation. *Mol. Neurobiol.* **2021**, *58*, 4506–4519, doi:10.1007/s12035-021-02405-x.
140. Huang, Y.; Zhu, L.; Li, H.; Ye, J.; Lin, N.; Chen, M.; Pan, D.; Chen, Z. Endometriosis Derived Exosomal miR-301a-3p Mediates Macrophage Polarization via Regulating PTEN-PI3K Axis. *Biomed. Pharmacother.* **2022**, *147*, 112680, doi:10.1016/j.biopha.2022.112680.
141. Liu, Y.; Li, X.; Zhang, Y.; Wang, H.; Rong, X.; Peng, J.; He, L.; Peng, Y. An miR-340-5p-Macrophage Feedback Loop Modulates the Progression and Tumor Microenvironment of Glioblastoma Multiforme. *Oncogene* **2019**, *38*, 7399–7415, doi:10.1038/s41388-019-0952-x.
142. Do, D.C.; Mu, J.; Zhou, Y.; Gao, P. miR-511-3p Limits Allergic Inflammation through M2 Macrophage Polarization and Modulating CCL2 Expression. *J. Allergy Clin. Immunol.* **2018**, *141*, AB80, doi:10.1016/j.jaci.2017.12.257.
143. Zhong, Y.; Yi, C. MicroRNA-720 Suppresses M2 Macrophage Polarization by Targeting GATA3. *Biosci. Rep.* **2016**, *36*, e00363, doi:10.1042/BSR20160105.
144. Qian, M.; Wang, S.; Guo, X.; Wang, J.; Zhang, Z.; Qiu, W.; Gao, X.; Chen, Z.; Xu, J.; Zhao, R.; et al. Hypoxic Glioma-Derived Exosomes Deliver microRNA-1246 to Induce M2 Macrophage Polarization by Targeting TERF2IP via the STAT3 and NF-κB Pathways. *Oncogene* **2020**, *39*, 428–442, doi:10.1038/s41388-019-0996-y.
145. Huang, S.; Liu, L.; Xu, Z.; Liu, X.; Wu, A.; Zhang, X.; Li, Z.; Li, S.; Li, Y.; Yuan, J.; et al. Exosomal miR-6733-5p Mediates Cross-Talk between Glioblastoma Stem Cells and Macrophages and Promotes Glioblastoma Multiform Progression Synergistically. *CNS Neurosci. Ther.* **2023**, *29*, 3756–3773, doi:10.1111/cns.14296.
146. Yang, Z.; Jiang, X.; Zhang, J.; Huang, X.; Zhang, X.; Wang, J.; Shi, H.; Yu, A. Let-7a Promotes Microglia M2 Polarization by Targeting CKIP-1 Following ICH. *Immunol. Lett.* **2018**, *202*, 1–7, doi:10.1016/j.imlet.2018.07.007.
147. Wang, Z.; Xu, L.; Hu, Y.; Huang, Y.; Zhang, Y.; Zheng, X.; Wang, S.; Wang, Y.; Yu, Y.; Zhang, M.; et al. miRNA Let-7b Modulates Macrophage Polarization and Enhances Tumor-Associated Macrophages to Promote Angiogenesis and Mobility in Prostate Cancer. *Sci. Rep.* **2016**, *6*, 25602, doi:10.1038/srep25602.
148. Xie, C.; Guo, Y.; Lou, S. LncRNA ANCR Promotes Invasion and Migration of Gastric Cancer by Regulating FoxO1 Expression to Inhibit Macrophage M1 Polarization. *Dig. Dis. Sci.* **2020**, *65*, 2863–2872, doi:10.1007/s10620-019-06019-1.
149. Liu, J.; Ding, D.; Jiang, Z.; Du, T.; Liu, J.; Kong, Z. Long Non-Coding RNA CCAT1/miR-148a/PKCζ Prevents Cell Migration of Prostate Cancer by Altering Macrophage Polarization. *The Prostate* **2019**, *79*, 105–112, doi:10.1002/pros.23716.
150. Ye, Y.; Xu, Y.; Lai, Y.; He, W.; Li, Y.; Wang, R.; Luo, X.; Chen, R.; Chen, T. Long Non-Coding RNA Cox-2 Prevents Immune Evasion and Metastasis of Hepatocellular Carcinoma by Altering M1/M2 Macrophage Polarization. *J. Cell. Biochem.* **2018**, *119*, 2951–2963, doi:10.1002/jcb.26509.
151. Sun, D.; Yu, Z.; Fang, X.; Liu, M.; Pu, Y.; Shao, Q.; Wang, D.; Zhao, X.; Huang, A.; Xiang, Z.; et al. LncRNA GAS5 Inhibits Microglial M2 Polarization and Exacerbates Demyelination. *EMBO Rep.* **2017**, *18*, 1801–1816, doi:10.15252/embr.201643668.
152. Li, Z.; Feng, C.; Guo, J.; Hu, X.; Xie, D. GNAS-AS1/miR-4319/NECAB3 Axis Promotes Migration and Invasion of Non-Small Cell Lung Cancer Cells by Altering Macrophage Polarization. *Funct. Integr. Genomics* **2020**, *20*, 17–28, doi:10.1007/s10142-019-00696-x.
153. Chen, J.; Gao, Y.; Zhong, J.; Wu, X.; Leng, Z.; Liu, M.; Wang, Y.; Wang, Y.; Yang, X.; Huang, N.; et al. Lnc-H19-Derived Protein Shapes the Immunosuppressive Microenvironment of Glioblastoma. *Cell Rep. Med.* **2024**, *5*, 101806, doi:10.1016/j.xcrm.2024.101806.
154. Wang, J.; Wang, N.; Zheng, Z.; Che, Y.; Suzuki, M.; Kano, S.; Lu, J.; Wang, P.; Sun, Y.; Homma, A. Exosomal lncRNA HOTAIR Induce Macrophages to M2 Polarization via PI3K/ p-AKT /AKT Pathway and Promote EMT

and Metastasis in Laryngeal Squamous Cell Carcinoma. *BMC Cancer* **2022**, *22*, 1208, doi:10.1186/s12885-022-10210-5.

155. Tian, X.; Wu, Y.; Yang, Y.; Wang, J.; Niu, M.; Gao, S.; Qin, T.; Bao, D. Long Noncoding RNA LINC00662 Promotes M2 Macrophage Polarization and Hepatocellular Carcinoma Progression via Activating Wnt/β-Catenin Signaling. *Mol. Oncol.* **2020**, *14*, 462–483, doi:10.1002/1878-0261.12606.
156. Zhou, L.; Tian, Y.; Guo, F.; Yu, B.; Li, J.; Xu, H.; Su, Z. LincRNA-P21 Knockdown Reversed Tumor-Associated Macrophages Function by Promoting MDM2 to Antagonize* P53 Activation and Alleviate Breast Cancer Development. *Cancer Immunol. Immunother.* **2020**, *69*, 835–846, doi:10.1007/s00262-020-02511-0.
157. Yang, D.; Liu, K.; Fan, L.; Liang, W.; Xu, T.; Jiang, W.; Lu, H.; Jiang, J.; Wang, C.; Li, G.; et al. LncRNA RP11-361F15.2 Promotes Osteosarcoma Tumorigenesis by Inhibiting M2-Like Polarization of Tumor-Associated Macrophages of CPEB4. *Cancer Lett.* **2020**, *473*, 33–49, doi:10.1016/j.canlet.2019.12.041.
158. Ahmad, I.; Naqvi, R.A.; Valverde, A.; Naqvi, A.R. LncRNA MALAT1/microRNA-30b Axis Regulates Macrophage Polarization and Function. *Front. Immunol.* **2023**, *14*, doi:10.3389/fimmu.2023.1214810.
159. Wei, H.; Wu, X.; Huang, L.; Long, C.; Lu, Q.; Huang, Z.; Huang, Y.; Li, W.; Pu, J. LncRNA MEG3 Reduces the Ratio of M2/M1 Macrophages Through the HuR/CCL5 Axis in Hepatocellular Carcinoma. *J. Hepatocell. Carcinoma* **2024**, *11*, 543–562, doi:10.2147/JHC.S449090.
160. Cao, J.; Dong, R.; Jiang, L.; Gong, Y.; Yuan, M.; You, J.; Meng, W.; Chen, Z.; Zhang, N.; Weng, Q.; et al. LncRNA-MM2P Identified as a Modulator of Macrophage M2 Polarization. *Cancer Immunol. Res.* **2019**, *7*, 292–305, doi:10.1158/2326-6066.CIR-18-0145.
161. Toker, J.; Iorgulescu, J.B.; Ling, A.L.; Villa, G.R.; Gadet, J.A.M.A.; Parida, L.; Getz, G.; Wu, C.J.; Reardon, D.A.; Chiocca, E.A.; et al. Clinical Importance of the lncRNA NEAT1 in Cancer Patients Treated with Immune Checkpoint Inhibitors. *Clin. Cancer Res.* **2023**, *29*, 2226–2238, doi:10.1158/1078-0432.CCR-22-3714.
162. Zhou, Y.; Zhao, W.; Mao, L.; Wang, Y.; Xia, L.; Cao, M.; Shen, J.; Chen, J. Long Non-Coding RNA NIFK-AS1 Inhibits M2 Polarization of Macrophages in Endometrial Cancer through Targeting miR-146a. *Int. J. Biochem. Cell Biol.* **2018**, *104*, 25–33, doi:10.1016/j.biocel.2018.08.017.
163. Liu, Y.; Shi, M.; He, X.; Cao, Y.; Liu, P.; Li, F.; Zou, S.; Wen, C.; Zhan, Q.; Xu, Z.; et al. LncRNA-PACERR Induces pro-Tumour Macrophages via Interacting with miR-671-3p and m6A-Reader IGF2BP2 in Pancreatic Ductal Adenocarcinoma. *J. Hematol. Oncol.J Hematol Oncol* **2022**, *15*, 52, doi:10.1186/s13045-022-01272-w.
164. Huang, L.; Wang, Z.; Liao, C.; Zhao, Z.; Gao, H.; Huang, R.; Chen, J.; Wu, F.; Zeng, F.; Zhang, Y.; et al. PVT1 Promotes Proliferation and Macrophage Immunosuppressive Polarization through STAT1 and CX3CL1 Regulation in Glioblastoma Multiforme. *CNS Neurosci. Ther.* **2024**, *30*, e14566, doi:10.1111/cns.14566.
165. Liang, Z.; Liu, H.; Wang, F.; Xiong, L.; Zhou, C.; Hu, T.; He, X.; Wu, X.; Xie, D.; Wu, X.; et al. LncRNA RPPH1 Promotes Colorectal Cancer Metastasis by Interacting with TUBB3 and by Promoting Exosomes-Mediated Macrophage M2 Polarization. *Cell Death Dis.* **2019**, *10*, 829, doi:10.1038/s41419-019-2077-0.
166. Li, X.; Lei, Y.; Wu, M.; Li, N. Regulation of Macrophage Activation and Polarization by HCC-Derived Exosomal lncRNA TUC339. *Int. J. Mol. Sci.* **2018**, *19*, 2958, doi:10.3390/ijms19102958.
167. Pi, L.; Fang, B.; Meng, X.; Qian, L. LncRNA XIST Accelerates Burn Wound Healing by Promoting M2 Macrophage Polarization through Targeting IL-33 via miR-19b. *Cell Death Discov.* **2022**, *8*, 220, doi:10.1038/s41420-022-00990-x.
168. Yang, J.; Gong, Z.; Dong, J.; Bi, H.; Wang, B.; Du, K.; Zhang, C.; Chen, L. lncRNA XIST Inhibition Promotes M2 Polarization of Microglial and Aggravates the Spinal Cord Injury via Regulating miR-124–3p / IRF1 Axis. *Heliyon* **2023**, *9*, e17852, doi:10.1016/j.heliyon.2023.e17852.
